# Effects of Wildflower Strips and an Adjacent Forest on Aphids and Their Natural Enemies in a Pea Field

**DOI:** 10.3390/insects8030099

**Published:** 2017-09-13

**Authors:** Séverin Hatt, Pierre Mouchon, Thomas Lopes, Frédéric Francis

**Affiliations:** 1 TERRA—AgricultureIsLife, Gembloux Agro-Bio Tech, University of Liège, Passage des Déportés 2, 5030 Gembloux, Belgium; 2 Functional and Evolutionary Entomology, Department of Agronomy, Biology and Chemistry, Gembloux Agro-Bio Tech, University of Liège, Passage des Déportés 2, 5030 Gembloux, Belgium; mouchonpierre@gmail.com (P.M.); tlopes@doct.ulg.ac.be (T.L.); frederic.francis@ulg.ac.be (F.F.); 3 Institut Supérieur d’Agriculture de Lille, 48 Boulevard Vauban, 59046 Lille, France

**Keywords:** conservation biological control, Syrphidae, Coccinellidae, parasitism, *Pisum sativum* L.

## Abstract

Landscape diversification is a key element for the development of sustainable agriculture. This study explores whether the implementation of habitats for pest natural enemies enhances conservation biological control in an adjacent field. In the present study conducted in Gembloux (Belgium) in 2016, the effect of two different habitats (wildflower strips and a forest) and aphid abundance on the density of aphid natural enemies, mummified aphids and parasitism on pea plants was assessed through visual observations. The effect of the habitats on aphids was also evaluated. The habitats but not aphid density significantly affected hoverfly larvae, which were more abundant adjacent to wildflower strips than to the forest. The contrary was observed for ladybeetle adults, which were positively related with aphids but not affected by the adjacent habitats. The abundance of mummies and the parasitism rate were significantly affected by both the habitats and aphid density. They were both significantly enhanced adjacent to wildflower strips compared to the forest, but the total parasitism rate was low (<1%), questioning whether parasitoids could significantly control aphids on the pea crop. As for the aphids, their abundance was not significantly affected by the adjacent habitats. These results are discussed with respect to the potential of these habitats to provide overwintering sites and food resources for natural enemies, and thereby enhance conservation biological control.

## 1. Introduction

The adoption of intensive agricultural practices in Europe has led to a simplification of agricultural landscapes and an increased use of external inputs, among others insecticides [1]. While crop yield has generally increased, negative side effects such as detrimental impacts on the environment and human health [2,3,4] ask for a transition towards more sustainable food systems [5]. Moreover, the resistances pests develop to insecticides [6], as well as the ever tighter regulation on their use in the European Union [7], encourage the development of non-chemically based practices. Among other strategies [8], conservation biological control (CBC) is the “use of tactics and approaches that involve the manipulation of the environment (i.e., the habitat) of natural enemies so as to enhance their survival, and/or physiological and behavioural performance, and resulting in enhanced effectiveness” [9]. Whereas crop fields are often simplified (i.e., sown with a single crop species) and disturbed (i.e., crop plants are harvested a few months after sowing), semi-natural habitats are diversified—and possibly permanent—areas able to provide to insects floral food resources, prey for predators and hosts for parasitoids, overwintering sites and shelters against disturbances [10]. Their implementation and management is subsidised in many European countries through agri-environmental policies, in order to “reduce environmental risks associated with modern farming on the one hand, and preserve nature and cultivated landscapes on the other hand” [11]. Recently, their potential benefits for CBC have been reviewed [12]. At the landscape scale, numerous studies have evaluated the potential of habitat diversity in enhancing CBC, mostly showing that increased landscape complexity enhances natural enemies and pest reduction (e.g., [13,14,15], but see [16]). At the local scale, wildflower strips (WFS) sown at field margins [17] or within fields [18] and hedgerows [19], as well as woodlots [20], adjacent to fields can also enhance CBC (but see [21]).

Aphids (Hemiptera: Aphididae) are a main pest of crops in temperate climate regions [22]. They can be harmful by sucking plant phloem, producing honeydew and transferring viruses [23]. Their insect natural enemies are predators and parasitoids. Alignier et al. [24] compared how different natural habitats (i.e., woodlots, grasslands, hedgerows) affect aphids, hoverflies (Diptera: Syrphidae) and parasitism across various spatial scales in wheat (*Triticum aestivum* L.) fields. Nevertheless, few studies to our knowledge have studied the effect of two different habitats surrounding a same field on the enhancement of CBC. Additionally, pest abundance may be a significant driver in the spread of natural enemies in fields [25]. By focusing on WFS and a forest, the present study aims at answering the following questions: (i) Do WFS and forests differently affect the abundance of aphids and their natural enemies, as well as parasitism, in the adjacent crop? (ii) Does aphid density—compared to habitats—drive natural enemy spread in fields?

## 2. Materials and Methods

### 2.1. Field Setup

**Figure 1:**
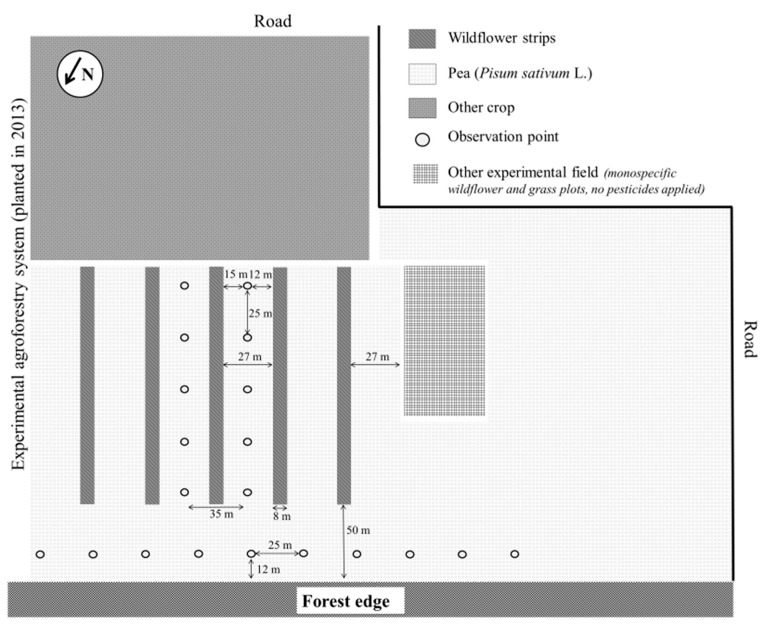
Experimental design.

This study was conducted at the experimental farm of Gembloux Agro-Bio Tech (University of Liège), Namur Province of Belgium (50°34′03″ N; 4°42′27″ E). On a surface of 9 ha, five replicated WFS (125 m × 8 m) were sown on 6 June 2013 at a distance of 27 m from each other (Figure 1). Each WFS was composed of 17 perennial wildflower species (Table S1, see [26] for more details about the sowing protocol) and three grass species, commonly found in Belgian grasslands and available on the market (seeds were obtained from ECOSEM, Belgium). A 5 ha forest was located at its northern side (i.e., the Escaille natural reserve) (Figure 1). Since their implementation in 2013, the WFS have been mown twice a year at the end of June and September. Winter pea (*Pisum sativum* L.) was sown on 15 November 2015. No insecticides or herbicides were used in the whole experimental area.

### 2.2. Insect Monitoring

Aphids (both alates and apterous), their insect natural enemies (i.e., ladybeetle adults and larvae [Coleoptera: Coccinellidae], hoverfly larvae [Diptera: Syrphidae] and lacewing larvae [Neuroptera: Chrysopidae]) as well as mummies (i.e., aphids parasitised by parasitoids [Hymenoptera]) were counted every week on pea plants between 9 May 2016 and 13 July 2016 (i.e., 10 weeks). Ten locations in the pea field in between WFS and 10 locations along the forest edge were chosen with a distance of 25 m between each, marked by a permanent stick. Every week, 20 pea plants were randomly chosen at each location to monitor the abundance of aphids, mummies and their natural enemies on the plants. Regarding peas in between WFS, the two central crop strips were considered as they presented a similar number of WFS on each of their sides. Five locations were chosen in each pea strip (Figure 1). Rainy days were avoided and no distinction between larval stages was made.

### 2.3. Statistical Analyses

First, Generalised Linear Mixed Models (GLMM, R function ‘glmer’, package ‘lme4’ [27]) with Poisson error distribution (log-link function) were fitted to test whether the habitat adjacent to peas (i.e., WFS vs. forest) and aphid abundance affected the density of natural enemies and mummies. Habitats and aphid abundance were analysed as fixed factors while the observation locations were included as random factors (as observations were made over several consecutive weeks at the same location) nested into the habitat effect (in order to integrate their dependent relationship, i.e., pseudo-replications).

Second, a GLMM with Poisson error distribution was fitted to test whether the habitats adjacent to peas affected aphid density. The habitats were considered fixed factors while the observation locations nested into the habitat effect were included as random factors.

However, data overdispersion was found on these GLMMs. Therefore, Generalised Linear Models with a negative binomial error distribution were fitted instead (R function ‘glm.nb’, package ‘MASS’ [28]), as suggested by Ver Hoef and Boveng [29]. The effects of fixed factors were tested using likelihood-ratio tests (*p* < 0.05).

Third, a Linear Mixed Model (LMM, R function ‘lmer’, package ‘lmerTest’ [30]) was fitted to test whether the habitats adjacent to peas and aphid abundance affected the parasitism rate, calculated as [mummies/(aphids + mummies)] × 100 [31]. The habitats and aphid abundance were analysed as fixed factors while the observation locations nested into the habitat effect were included as random factors. Parasitism rate and aphid abundance were log10(x + 1)-transformed prior to the analyses. The effects of fixed factors were tested using an analysis of variance (ANOVA) (*p* < 0.05). All the statistical analyses were performed using R Core Team [32].

## 3. Results

**Figure 2:**
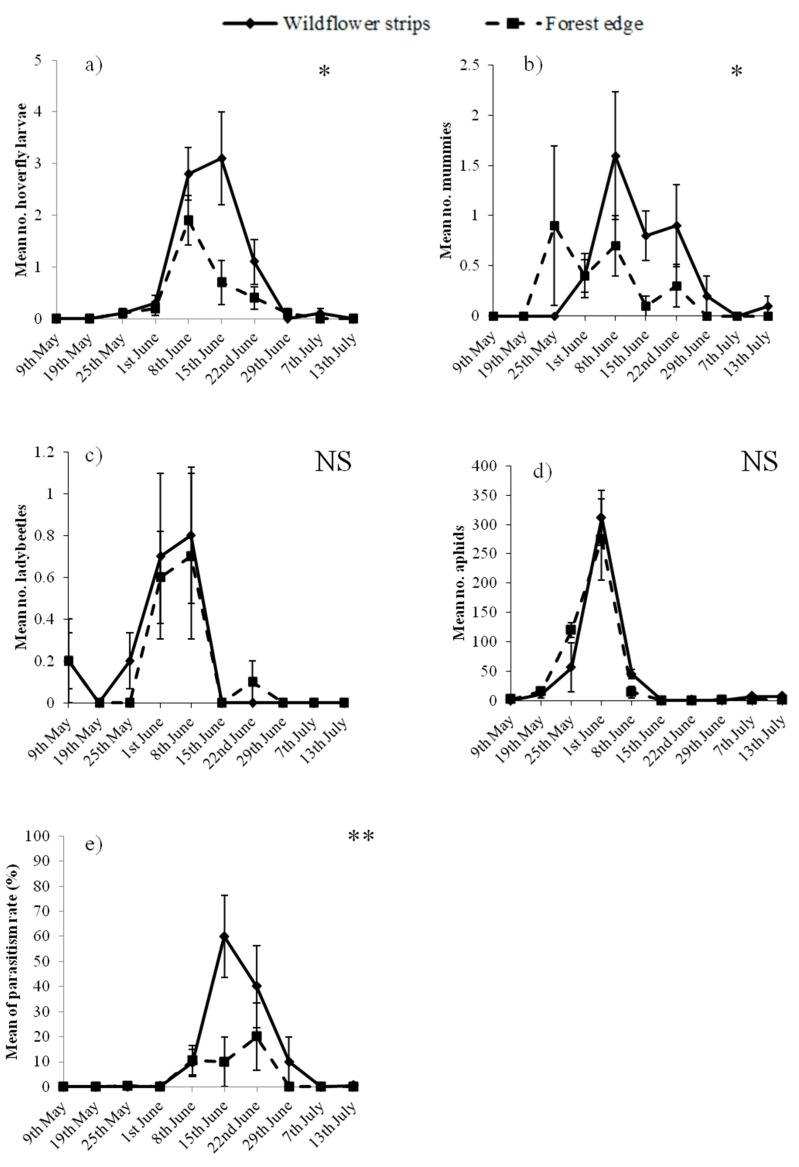
Mean (±SEM) abundance of (**a**) hoverfly larvae; (**b**) mummies; (**c**) ladybeetle adults; (**d**) aphids and (**e**) parasitism rate (%), through time on pea plants adjacent to wildflower strips (continuous line) and the forest edge (dotted line). * *p* < 0.05; ** *p* < 0.01; NS: not significant.

In total, 8721 aphids, 109 hoverfly larvae, 35 ladybeetle adults and 64 mummies, but no ladybeetle or lacewing larvae were observed on pea plants. Habitats, but not aphid abundance, significantly affected hoverfly larvae, which were more abundant adjacent to WFS than to the forest edge (Table 1 and Figure 2a). The opposite was observed for ladybeetle adults, which were positively correlated with aphids but not affected by the adjacent habitats (Table 1 and Figure 2c). Concerning mummified aphids, their abundance was significantly affected by both habitats and aphid abundance, since they increased with the abundance of aphids and were generally more abundant adjacent to WFS than the forest (Table 1 and Figure 2b). Despite the aphid density on pea plants not being affected by the habitats (Table 1 and Figure 2d), the parasitism rate was higher adjacent to WFS (Table 2 and Figure 2e), and was negatively correlated with aphids.

## 4. Discussion

### 4.1. Aphid Predators

Among aphid predators, hoverfly larvae but not ladybeetle adults counted on pea plants were affected by the type of adjacent habitats, being more abundant adjacent to WFS. The present results confirm previous observations that WFS sown within fields significantly enhance the presence of aphidophagous hoverflies [18]. Alignier et al. [24] previously reported that fields (i.e., wheat fields in this case) bordered by woodlots are colonised early by aphidophagous hoverfly larvae, suggesting like Sarthou et al. [33] that hoverflies use woodlots for overwintering. Thus, despite a potential overwintering of hoverflies in the forest, hoverflies were attracted early by the flower strips. Moreover, the non-significant relation between hoverfly larvae and aphid abundances shows that WFS, more than the presence of prey, determined the distribution of hoverfly larvae. Hoverfly adults feed exclusively on floral nectar and pollen, providing them energy and proteins that are essential for their longevity and reproduction, respectively [34]. The search for aphids by adults for laying their eggs is a second step [35], which is not the case for ladybeetles, whose adults and larvae can feed on both flowers and prey [34]. In the present study, prey more than habitats have affected ladybeetle distribution. According to Lundgren [36], floral resources can be essential for ladybeetles when prey are scarce; however, in the present case aphids were abundant (on average: 249 aphids for one ladybeetle), which can explain why flowers did not significantly attract ladybeetles.

### 4.2. Aphid Parasitism

The abundance of mummies and parasitism rate were significantly affected by both habitats adjacent to the peas and aphid density. While an increased density of aphids was related to an increased number of mummies (Table 1), it did not lead to an increased parasitism rate (Table 2). This suggests that aphid density rose more quickly than the number of mummies; consequently, parasitoids were not able to stop aphid development. This finding follows Thies et al. [16], who suggested that parasitoids would be able to control aphids only in a situation of low aphid density, which was not the case here. Figure 2b shows that mummies were more numerous adjacent to the forest edge compared to WFS in May, while the contrary was observed from June. One explanation is that aphids were slightly more abundant adjacent to the forest edge in May (Figure 2d), which could have attracted the parasitoids (the number of mummies was generally positively correlated with aphid density, Table 1). Another reason is that parasitoids may have overwintered in the forest rather than in the WFS. For Thies et al. [16], any permanent habitat could be used as an overwintering site by parasitoids; however, according to Sarthou et al. [37] parasitoids preferentially overwinter in grassy strips rather than forests. Nevertheless, in the present experiment, WFS were mown the previous autumn (i.e., September, see Materials and Methods), leading to a less diverse habitat than that of the Escaille natural reserve. A botanical survey conducted in the natural reserve reported a diversity of plant species which were not mown before winter [38]. The significantly higher parasitism rate, and the higher abundance of mummies from June, in the crop adjacent to WFS, however, indicate that once grown and blooming, flowers can strongly attract parasitoids. Indeed, floral nectar is an essential energy resource for parasitoids [39]. Previous studies measuring aphid parasitism adjacent to grassy habitats reported non-significant effects [17,24] while a negative effect of woodlots was found [24]. In the present study, while a high diversity of flowering species was found in the forested reserve [38], their probable relatively lower density, compared to sown WFS, may explain the lower parasitism at its edge in summer.

### 4.3. Perspectives

As aphid abundance was not affected by the adjacent habitat (Table 1), no difference in effect on enhancing pest control was shown between WFS and the forest. However, the present results indicate that flowering habitats such as WFS can support hoverflies and enhance the parasitism rate, while permanently vegetated ones, such as a forest, may offer overwintering sites to parasitoids, potentially allowing early aphid parasitism in the adjacent crop. Therefore, these two types of habitats show potential synergies that may benefit natural enemies when implemented close to one another.

As the study was conducted over a single season in one pea field, the preliminary results obtained here should not be generalized and further research is required to confirm them. In addition, conducting the study over multiple years and multiplying the experimental sites would also allow avoiding pseudo-replications within the treatments. Regarding insects, considering adults of hoverflies and parasitoids through insect trapping would help specify the behaviour of aphid natural enemies in the pea field.

Finally for reducing pest density, combining the implementation of semi-natural habitats for natural enemies (top-down control), with within field practices that increase crop diversity, such as inter- or cover-cropping (bottom-up control), should be considered [40,41]. Indeed, diversified cropping systems may complicate the ability of pests to find and establish on their host plants, in the end limiting their development [42]. The present observations may encourage considering several permanent natural habitats within fields and at margins for potentially enhancing synergies to support natural enemies in agricultural landscapes.

## Figures and Tables

**Table 1:** Effect of habitats (wildflower strips vs. forest) and aphid abundance, as well as their interaction, on the abundance of aphid natural enemies and mummified aphids; and effect of habitats on aphid abundance only. Degrees of freedom (df), χ^2^-values and *p*-values of likelihood ratio tests performed on generalised linear models are given. The effect signs related to aphid effects are retrieved from the estimates of models when significant. * *p* < 0.05; *** *p* < 0.001.

Hoverfly Larvae	df	χ²	*p*-Value	Effect
Habitat	1	5.01	0.025 *	
Aphid	1	1.13	0.288	
Habitat: Aphid	1	0.038	0.845	
**Ladybeetle adults**				
Habitat	1	0.11	0.744	
Aphid	1	11.9	<0.001 ***	(+)
Habitat: Aphid	1	0.026	0.871	
**Mummified aphids**				
Habitat	1	4.3	0.038 *	
Aphid	1	8.7	0.003 **	(+)
Habitat: Aphid	1	4.47	0.034 *	
**Aphids**				
Habitat	1	0.002	0.962	

**Table 2:** Effect of habitats (wildflower strips vs. forest) and aphid abundance, as well as their interaction, on parasitism rate calculated as [mummies/(mummies + aphids)] × 100. Degrees of freedom (df), F-values and *p*-values of ANOVA performed on the linear mixed model are given. The effect sign related to aphid effect is retrieved from the estimate of the model. * *p* < 0.05; ** *p* < 0.01; *** *p* < 0.001.

Parasitism Rate	df	F	*p*-Value	Effect
Habitat	1	10.6	0.001 **	
Aphid	1	12	<0.001 ***	(−)
Habitat: Aphid	1	4.71	0.031 *	

## Supplementary Materials

The following are available online at www.mdpi.com/2075-4450/8/3/99/s1. Table S1. Perennial flowering and grassy species sown in each wildflower strip in 2013.
