# Supplementary material for: Effects of Wildflower Strips and an Adjacent Forest on Aphids and Their Natural Enemies in a Pea Field
Source: Insects. 2017 Sep 13;8(3):99. doi: 10.3390/insects8030099 (PMC5620719; doi:10.3390/insects8030099)
Supplement: Supplementary file 1 [file insects-08-00099-s001.pdf]

Table S1. Perennial flowering and grassy species sown in each wildflower strip in 2013.

Family	Species	Density (kg/ha)
	**Flowering species**	
Apiaceae	*Anthriscus sylvestris* (L.) Hoffm.	0.3
Apiaceae	*Heracleum sphondylium* (L.)	0.1
Asteraceae	*Achillea millefolium* (L.)	0.4
Asteraceae	*Crepis biennis* (L.)	0.1
Asteraceae	*Hypochaeris radicata* (L.)	0.1
Asteraceae	*Leontodon hispidus* (L.)	0.2
Asteraceae	*Leucanthemum vulgare* Lam.	0.2
Dipsacaceae	*Knautia arvensis* (L.) Coulter	0.2
Fabaceae	*Lotus corniculatus* (L.)	0.1
Fabaceae	*Medicago lupulina* (L.)	0.1
Fabaceae	*Trifolium pratense* (L.)	0.1
Geraniaceae	*Geranium pyrenaicum* Burm. fil.	0.1
Lamiaceae	*Origanum vulgare* (L.)	0.1
Lamiaceae	*Prunella vulgaris* (L.)	0.2
Lythraceae	*Lythrum salicaria* (L.)	0.2
Malvaceae	*Malva moschata* (L.)	0.1
Rubiaceae	*Galium verum* (L.)	0.2
	**Grass species**	
Poaceae	*Agrostis* spp. (L.)	5
Poaceae	*Festuca rubra* (L.)	11.5
Poaceae	*Poa pratensis* (L.)	5
